# Effects of Simulated Visual Impairment Conditions on Movement and Anxiety during Gap Crossing

**DOI:** 10.3390/healthcare12010042

**Published:** 2023-12-24

**Authors:** Tadashi Uno, Taihei Matsuo, Masanari Asano, Ping Yeap Loh

**Affiliations:** 1 Center of Liberal Arts and Science, Sanyo-Onoda City University, Yamaguchi 756-0884, Japan; 2 Graduate School of Design, Kyushu University, Fukuoka 815-8540, Japan; matsuo.taihei.273@s.kyushu-u.ac.jp; 3 Faculty of Humanity-Oriented Science and Engineering, Kindai University, Fukuoka 820-8555, Japan; asano@fuk.kindai.ac.jp; 4 Department of Life Design and Science, Faculty of Design, Kyushu University, Fukuoka 815-8540, Japan; py-loh@design.kyushu-u.ac.jp

**Keywords:** platform transition, environmental adaptation, visually impaired navigation, tunnel vision, State-Trait Anxiety Inventory, orientation and mobility

## Abstract

This study investigated the effects of visual conditions associated with progressive eye disease on movement patterns and anxiety levels during gap-crossing tasks. Notably, 15 healthy young adults performed crossover platforms with a 10 cm gap at three different heights, namely equal (0 cm), raised (+15 cm), and lowered (−15 cm) levels, under four vision conditions, namely normal or corrected eyesight, 10° tunnel vision, 5° tunnel vision, and 5° tunnel vision with 0.04 occlusion. Leg movements during gap crossing were analyzed using three-dimensional motion analysis. The results highlighted a distinct motion pattern in the trajectories of participants’ legs under the different visual conditions. Specifically, at the point where the gap-crossing movement began (D1), the normal or corrected eyesight conditions resulted in further separation between the steps compared with the other visual conditions. The highest point of the foot during movement (D2) did not differ between the visual conditions, except for the 0 cm step. Furthermore, anxiety levels, as quantified by the State-Trait Anxiety Inventory (STAI-S) questionnaire, were exacerbated under conditions of restricted visual information. In conclusion, visual impairments associated with progressive ocular diseases may perturb complex motor movement patterns, including those involved in gap-crossing tasks, with heightened anxiety potentially amplifying these disturbances.

## 1. Introduction

As sociodemographic status and life expectancy increase, more people are living into adulthood. This results in an older average population and a shift in disease burden toward non-communicable diseases and disabilities in many countries worldwide [1,2,3]. This epidemiological transition also affects many ocular diseases, escalating personal and societal costs [4]. A systematic review and meta-analysis of population-based surveys conducted from 1980 to 2018 provided projections of visual impairment by 2050. These projections estimate that approximately 61 million people will be blind, approximately 474 million will have moderate-to-severe visual impairment, approximately 360 million will experience mild visual impairment, and approximately 866 million will be affected by uncorrected presbyopia [5]. Moreover, the diseases that cause visual impairment and their onset vary between individuals and regions [3].

These epidemiological challenges are particularly relevant in countries such as Japan, which has a high rate of aging. In these nations, there is an increasing demand for comprehensive low-vision care, covering not only medical treatment, but also educational, vocational, welfare, and daily life support. Among these, the concept of orientation and mobility (O&M) is becoming increasingly vital for ensuring safe mobility and behavior in individuals with visual impairments [6]. This necessity is underscored by the challenges faced by individuals with progressive eye diseases, such as retinitis pigmentosa, which lead to a gradual loss of visual acuity and significantly affect daily functioning and overall well-being. Impaired vision, which is known to compromise spatial awareness, heightens the risk of accidents and falls [7,8,9,10] due to deficits in depth perception and contrast sensitivity, thereby making tasks such as gap crossing challenging. When visual cues are present, individuals can accurately evaluate gaps and devise safe and energy-efficient plans [11]. However, without visual input, reliance shifts to other sensory data and prior environmental knowledge, potentially leading to suboptimal strategies. This highlights the critical role of visual perception in obstacle navigation. Previous studies have highlighted the importance of visual perception in navigating various environmental challenges, and how impaired vision can disrupt motor tasks [12,13,14,15]. In addition, studies have analyzed adaptive gaits in the daily lives of visually impaired people, such as obstacle avoidance [16] and stair climbing [17,18]. However, there is a distinct lack of comprehensive research on the correlation between environmental factors and visual impairment, particularly those that significantly contribute to fall incidents, such as gaps. Furthermore, vision loss is associated with the fear of falling [19,20], and studies have identified visual field loss and decreased contrast sensitivity as the primary visual predictors of the fear of falling in patients with glaucoma and age-related macular degeneration, respectively [21,22]. In other words, visually impaired individuals tend to experience more psychological anxiety about mobility than individuals with normal vision, which is expected to have a significant effect on their movement patterns. Consequently, the impact of visual limitations on psychological anxiety during movement must be assessed.

Gap-crossing movements involve the harmonious integration of visual perception and motor coordination; thus, they are categorized as intricate motor tasks. However, the specific psychological and motor challenges encountered during gap crossing under visual constraints remain elusive. This study aimed to investigate the effects of visual conditions, particularly those associated with progressive eye disease, on movement patterns and anxiety levels during gap-crossing tasks. Such research is essential for developing interventions targeted at enhancing mobility and reducing anxiety in people with progressive eye diseases, thereby contributing significantly to the field of O&M and improving the quality of life of those with visual impairments.

## 2. Materials and Methods

### 2.1. Participants

Fifteen healthy young adults with normal or corrected vision were recruited from a university community (Table 1). None of the participants had musculoskeletal or visual impairments that could affect their mobility. Participants were informed orally and in writing about the experiment’s purpose, method, risks, and concerns, as well as the protection of their anonymity, and their written consent was obtained. The Ethics Committee of the Faculty of Design at Kyushu University approved this study (Approval No. 475).

We obtained the sample size suggestion (*n* = 18) by G*Power (3.1.9.6) for repeated measures ANOVA with an effect size of f = 0.3, alpha of 0.05, and power of 0.80. Post hoc power analysis revealed a sample size of 15 given a power of 0.75 with an effect size of f = 0.3 and an alpha of 0.05.

### 2.2. Experimental Environment

The experimental space was free of noise and intruding light. The illuminance, measured using a digital illuminance meter (SHINWA, Niigata, Japan), was 1100 lx on the platform and 1250 lx near the participant’s head.

**Figure 1:**
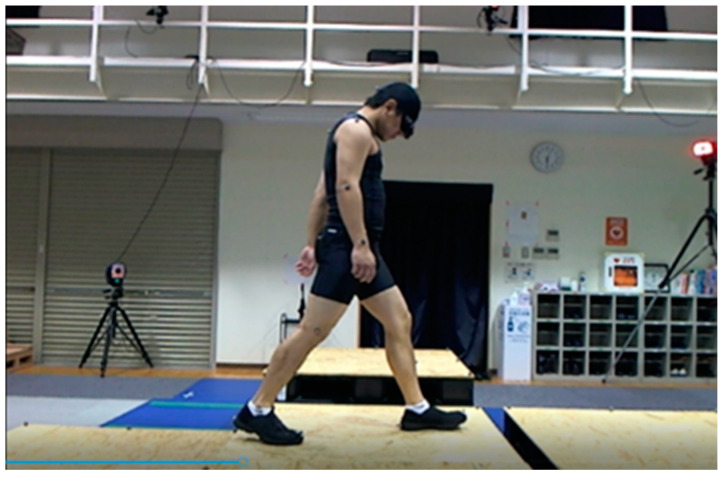
Experimental environment for gap crossing.

This study used a repeated measures design consisting of baseline and post-experiment questionnaire collection periods. The dependent variables were the three platforms and four vision conditions. Participants were instructed to walk and cross over platforms with a gap (10 cm) at the equal level (0 cm), raised platform (+15 cm), and lowered a platform (−15 cm) under four vision conditions: normal or corrected eyesight, 10° tunnel vision, 5° tunnel vision, and 5° tunnel vision with 0.04 occlusion. Simulation goggles (M. TAKATAOPTICAL Co., Ltd., Chiyoda, Japan) were used to reproduce the vision conditions. The dimensions of the origin platform were 1.1 m (L) × 1.1 m (W) × 0.3 m (H), and the three target platforms were 1.1 m × 1.1 m × 0.3 m (equal level, 0 cm step), 1.1 m × 1.1 m × 0.45 m (raised, +15 cm step), and 1.1 m × 1.1 m × 0.15 m (lowered, −15 cm step). A 10 cm gap was designated between the platforms. The platform was made of black plastic, and the walking surface was covered with ochre colored OSB boards (11 mm) (Figure 1).

### 2.3. Experimental Procedure

Before the trial, the dominant legs of all participants were confirmed using the dominant leg test of Waterloo Footedness Questionnaire—Revised (WFQ-R) [23]. Participants walked in shoes and wore tight-fitting black clothing and caps during the experiment. Before taking any measurements, participants spent sufficient time familiarizing themselves with each visual condition. Participants performed practice trials to ensure that they could follow the instructions and safely perform the tasks. The gap-crossing motion began and ended at each participant’s discretion. The order of the trial conditions in this study was randomized for each participant. Each condition had three successful trials.

Participants completed a standardized questionnaire before and after the procedure. This included standardized anxiety score: the State-Trait Anxiety Inventory Scale-5 questionnaire (STAIS-5; Table 2) and the State-Trait Anxiety Inventory Trait-5 questionnaire (STAIT-5; Table 3). STAIS-5 and STAIT-5 are extensively used anxiety measurement instruments with high degrees of sensitivity and validity for evaluating anxiety in clinical and procedural settings [24]. Items are rated on a 4-point scale from “not at all” to “very much”, with a maximum score of 20 for the highest levels of anxiety in the short, 5-question form. STAIT-5 was administered before the experiment, and STAIS-5 was completed after the first trial of each visual condition for each step.

### 2.4. Data Acquisition, Data Processing, and Analysis

**Figure 2:**
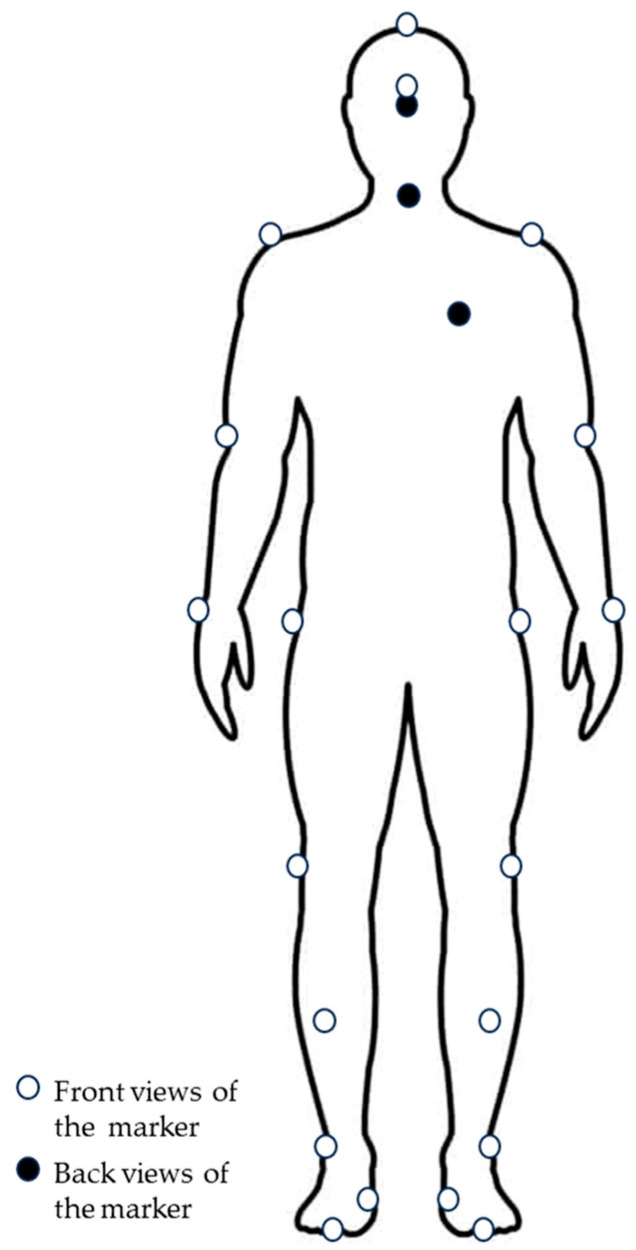
Helen-Hayes marker arrangement used in this study.

Cortex™ 3D motion analysis software (v7.0.0.1802), consisting of 9 infrared cameras (Motion Analysis Corporation, Santa Rosa, CA, USA) and 9.5 mm diameter reflective markers was used to record the participants’ motion at 100 Hz. The motion data were filtered using a 6 Hz Butterworth low-pass filter prior export for kinematic parameter analysis. Twenty-three reflective markers (Figure 2) were attached to the bony landmarks of participants’ bodies, as follows: head (fore, upper, and rear), upper limbs (acromion of shoulders, medial epicondyle of elbows, and ulnar styloid process of wrists), vertebral (7th cervical), and lower limbs (greater trochanter, lateral and medial epicondyle of femurs, shanks (center point between the knee joint and ankle joint), lateral and medial malleolus, calcaneus of the ankle, and 1st and 5th metatarsophalangeal joints).

**Figure 3:**
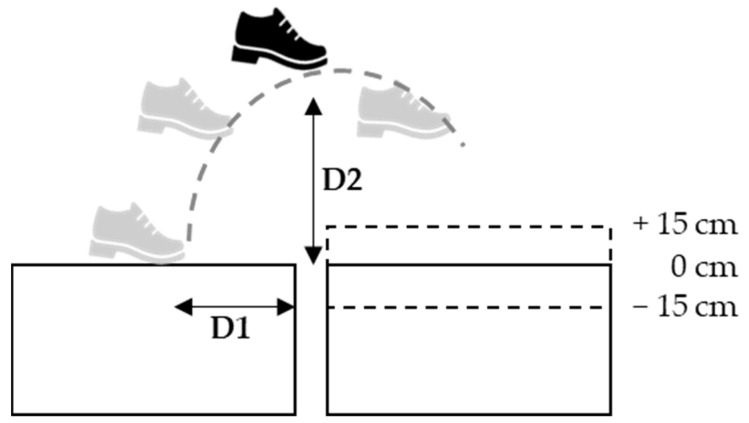
Analysis point of gap crossing. D1: Initiation of the step-over motion. D2: Highest point of the whole trajectory.

The average of three successful complete trials for each visual condition was analyzed using Kinema Tracer-Light (R5.0.3.2007) (Kissei Comtec, Nagano, Japan). The kinematic parameters of the leading foot (LF) and trailing foot (TF), such as the highest point of toe clearance, were calculated using the KineAnalyzer software (Kissei Comtec Co., Ltd., Nagano, Japan). The crossing phases were calculated using timestamps of the following crossing events: leading foot toe-off, leading foot initial contact, trailing foot toe-off, and trailing foot initial contact. These events were identified using the motion of the first metatarsophalangeal joint and ankle calcaneus markers. This study analyzed LF and TF during the crossing movement for the step distance (D1) and the highest point of the entire trajectory (D2) (Figure 3), where D1 was defined as the distance between the point where the toe marker left the ground and the gap.

### 2.5. Statistical Analysis

Statistical analyses were performed using IBM SPSS Version 26.0 (Chicago, IL, USA). The Shapiro–Wilk test confirmed the normality of all data, and Mauchly’s test was conducted to determine if the sphericity of the data had been violated. A one-way repeated-measures analysis of variance (ANOVA) was used to investigate the effects of vision conditions (normal or corrected eyesight, 10° tunnel vision, 5° tunnel vision, and 5° tunnel vision with 0.04 occlusion) on kinematic parameters. To analyze the effects of vision conditions on anxiety, one-way repeated-measures ANOVAs were performed, and anxiety was assessed using the STAIT-5 and STAIS-5. Post hoc analyses were conducted using the Bonferroni adjustment for multiple comparisons. The significance level was set at *p* = 0.05. All results are presented as means and standard deviation (SD).

## 3. Results

### 3.1. The Effects of Visual Conditions to Movement during Gap Crossing

**Figure 4:**
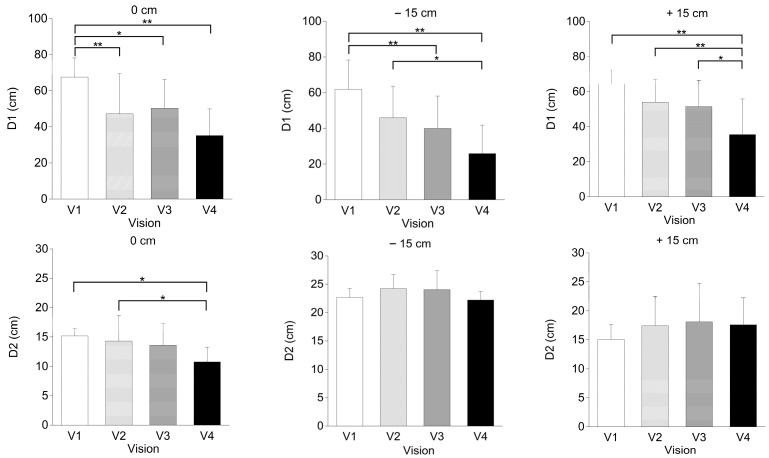
D1 (initiation of the step-over motion) and D2 (highest point of the whole trajectory) of the leading foot for the gaps. * *p* < 0.05, ** *p* < 0.01.

As depicted in Figure 4, a one-way repeated-measures ANOVA was performed. In the D1 phase, the V1 condition was significantly higher than the other visual conditions at the 0 cm step (*p* < 0.01, η^2^= 0.34), at the −15 cm step (*p* < 0.01, η^2^= 0.38), and at the +15 cm step (*p* < 0.01, η^2^= 0.34). During the D2 phase, the V4 condition was significantly lower than the V1 (*p* < 0.05, η^2^= 0.80) and V2 (*p* < 0.05, η^2^= 0.50) conditions for the 0 cm step. In contrast, there were no differences between the visual conditions for the other step conditions.

### 3.2. The Effects of Visual Conditions to Anxiety

The mean STAIT-5 score was 13.6 ± 1.9. The detailed STAIS-5 responses of the participants are presented in Table 4. The mean STAIS-5 value for each visual condition for the 0 cm step was significantly higher for V4 than for V1 (*p* < 0.01, η^2^ = 0.72) or V2 (*p* < 0.05, η^2^ = 0.42). A similar trend was observed for the +15 cm step. Furthermore, at the −15 cm step, V4 (*p* < 0.01, η^2^ = 0.81) and V3 (*p* < 0.05, η^2^ = 0.54) were significantly higher than V1. The detailed results for each of the STAIS-5 questions showed that the differences between the visual conditions were particularly pronounced for Items 1 and 2. Item 5 showed a marked difference between the visual conditions for the −15 cm and +15 cm step differences. In contrast, Item 4 showed no difference between the visual conditions in either step.

## 4. Discussion

This study investigated the effects of four visual conditions during three different gap-crossing tasks. As illustrated in Figure 4, visual conditions significantly affected the D1 and D2 phases of the gap-crossing movements at different platform settings. In the D1 phase, the V4 condition exhibited a significantly lower value than the other visual conditions across all of the platform conditions. The values in D1 consistently demonstrated that V1 was the furthest from the gap under all step conditions.

Visual information regarding the environment during locomotion allows participants to adjust their heading direction, avoid obstacles, and accommodate different surfaces [25]. The central fossa (approximately 1° eccentricity) and paracentral fossa (approximately 4–5° eccentricity) [26], which have a significantly high rod cell density within the visual field, are particularly important locations for the precise localization of the gap. It is impossible to always fix the most relevant visual information at the right time; our environment sometimes changes unpredictably, and the relevant information may not be localized to a single location [27]. In this case, gathering information through the peripheral visual field (outside the central fossa) is essential [26], as evidenced by clinical cases of mobility difficulties related to peripheral visual field defects in patient with retinitis pigmentosa [28,29,30].

In this study, participants’ peripheral vision was restricted to V2 (10° tunnel vision) and V3 (5° tunnel vision), and both eyes experienced afferent visual field constriction. Furthermore, in the V4 condition (5° tunnel vision with 0.04° occlusion), visual impairment was superimposed on the retained visual field. The results suggested that participants were able to perceive the gap’s position by utilizing their central vision in all visual conditions, as they initiated the straddling motion from a position close to the gap, ranging from V1 to V4. The brightness ratio between the floor and gap was also important for enabling participants to discern the gap. Notably, the brightness ratio is particularly significant for individuals with visual impairment because it enhances object perception precision [31]. The platform had an ochre in the experimental setting, whereas the gap was black, resulting in a sufficiently high brightness ratio, even under V4 conditions. Therefore, it could be inferred that participants were able to move closer to the gap.

In contrast, the D2 phase revealed no notable distinctions between the visual conditions for both the −15 cm and +15 cm steps. A similar pattern was observed at the 0 cm step, with limited differences between visual conditions. Previous studies have consistently reported that simulated visual impairments and visually impaired participants lead to increased toe clearance of LF when navigating obstacles or steps [17,18,32,33], which is a strategy adopted to minimize the risk of tripping [34]. In line with these findings, our study participants also raised their feet higher than usual to clear the gap, regardless of the visual condition. This behavior can be attributed to perspective changes stemming from the contraction of the afferent visual field. Individuals assess the distance between themselves and their surroundings, as well as the spatial relationship between objects, by maintaining specific body parts or objects within their visual field. However, when the effective visual field is severely limited, such as in the V3 and V4 conditions, obtaining detailed information on step heights from visual cues may be challenging. Consequently, participants safely cleared the gap by raising their feet to a certain height, irrespective of their visual status.

In the current study, a short version of the Spielberger STAI [24] was used to quantify participants’ anxiety resulting from visual field and vision limitations. Anxiety was categorized as state (STAI-S) or trait (STAI-T). Participants rated on how accurately each of the presented statements described their current emotional state using a 4-point Likert-type scale (1 = not at all, 4 = very much). The responses to the five items were then averaged to create an index of state anxiety, with higher values indicating an increased experience of arousal and tension in the present moment. State anxiety is characterized by increased anxiety, tension, and heart rate [35]. Previous research on older adults and clinical populations has demonstrated that state anxiety can significantly affect postural control [36,37] and gait [38], often leading to more conservative and restrictive movement patterns. To date, there have been no reports on the impact of the gap environment and visual constraints on state anxiety features. In this study, participants’ state anxiety levels notably increased with the intensification of the afferent visual field narrowing condition. This trend was particularly pronounced for the +15 cm and −15 cm steps in the V4 condition, where the field of view and visual acuity were restricted. While we found no prior research evaluating the influence of visual limitations on STAI scores during gap-crossing movements, our results align with those of studies highlighting the effects of visual field loss on mental health [39,40,41,42]. Similar trends were observed in a study that examined the effects of anxiety caused by falls and dual tasks on walking and obstacle avoidance movements [43,44]. Thus, amplified psychological anxiety significantly influences the inhibitory and destabilizing effects of foot-raising actions during step avoidance.

This study on the interplay between visual conditions, anxiety, and gap-crossing movements has several limitations. While focusing on specific visual conditions, it does not encompass all visual impairments. The pivotal role that we attributed to brightness ratios in discerning gaps did not account for potential external environmental influences. The abbreviated Spielberger STAI used may have understated participants’ anxiety levels. Additionally, the lack of comparable research on gap environments and state anxiety limits the correlation with previous studies. Moreover, participants had ample time beforehand for acclimatization, potentially leading to more deliberate and predictive movements, which could have impacted the results.

## 5. Conclusions

By evaluating varying visual conditions in gap-crossing activities, this study revealed that participants leveraged their central vision and the environment’s brightness contrasts to discern gaps, even under restricted visual conditions. The strategy of adaptively using visual information to determine the starting position of the gap-crossing motion was pronounced in the D1 phase. In contrast, foot height during locomotion did not differ significantly between visual conditions. Furthermore, state anxiety significantly increased when visual field restriction was combined with visual acuity loss, indicating that psychological factors had a significant impact on motor strategies, particularly during gap crossing. Future studies should broaden the scope of the visual conditions assessed and explore the role of psychological elements such as state anxiety in shaping movement behaviors. Moreover, we believe that conducting empirical studies on individuals with actual visual impairments and public facilities will ascertain the breadth of knowledge attainable through simulation-based methodologies. It is anticipated that integrating these findings into practical environments, such as pavement designs, will enhance the safety and accessibility for individuals with visual impairments. It is anticipated that integrating these findings into practical environments will further enhance safety and accessibility for individuals with visual impairments.

## Figures and Tables

**Table 1:** Characteristics of participants.

Characteristics Data	Participants (*n* = 15)	Male (*n* = 8)	Female (*n* = 7)
Age (year)	22.9 ± 1.3	22.4 ± 0.9	23.4 ± 1.6
Mass (kg)	61.6 ± 10.2	65.2 ± 8.2	55.0 ± 8.4
Height (m)	1.69 ± 0.09	1.77 ± 0.04	1.61 ± 0.05
BMI (kg/m^2^)	21.5 ± 2.7	21.7 ± 3.3	21.2 ± 2.1
* Leg length (cm)	86.7 ± 6.7	91.6 ± 4.7	82.3 ± 6.0
STAIT-5 (mean)	13.6 ± 1.9	13.8 ± 2.5	13.4 ± 1.0

* Leg length is defined as the distance from the greater trochanter to the lateral malleolus.

**Table 2:** State-Trait Anxiety Inventory Scale-5 (STAIS-5).

Item No.	Statements	Not at All	Somewhat	Moderately So	Very Much So
1	I feel upset	1	2	3	4
2	I feel frightened	1	2	3	4
3	I feel nervous	1	2	3	4
4	I am jittery	1	2	3	4
5	I feel confused	1	2	3	4

**Table 3:** State-Trait Anxiety Inventory Trait-5 (STAIT-5).

Item No.	Statements	Not at All	Somewhat	Moderately So	Very Much So
1	I feel that difficulties are piling up so that I cannot overcome them.	1	2	3	4
2	I worry too much over something that really does not matter.	1	2	3	4
3	Some unimportant thoughts run through my mind and bothers me.	1	2	3	4
4	I take disappointments so keenly that I cannot put them out of my mind.	1	2	3	4
5	I get in a state of tension or turmoil as I think over my recent concerns and interests.	1	2	3	4

**Table 4:** Effect of visual conditions for the STAIS-5 scores (mean ± SD). * *p* < 0.05, ** *p* < 0.01.

Platform	Item No.	V1	V2	V3	V4	F	*p*-Value
0 cm	1	1 ± 0	1.27 ± 0.46	1.27 ± 0.46	1.93 ± 0.89	7.93	** V1 < V4, ** V2 < V4, ** V3 < V4
	2	1 ± 0	1.67 ± 0.62	1.53 ± 0.64	2.33 ± 0.82	12.38	* V1 < V2, ** V1 < V4, * V2 < V4, ** V3 < V4
	3	1 ± 0	1.33 ± 0.62	1.6 ± 0.91	1.73 ± 0.7	3.69	* V1 < V4
	4	1 ± 0	1.13 ± 0.35	1.2 ± 0.41	1.33 ± 0.82	1.2	ns
	5	1 ± 0	1.53 ± 0.83	1.53 ± 0.64	2 ± 0.93	5.11	** V1 < V4
	Total	5 ± 0	6.93 ± 2.25	7.13 ± 2.56	9.33 ± 3.06	8.98	** V1 < V4, * V2 < V4
−15 cm	1	1 ± 0	1.27 ± 0.46	1.33 ± 0.49	2.2 ± 1.15	9.22	** V1 < V4, ** V2 < V4, ** V3 < V4
	2	1 ± 0	1.53 ± 0.64	1.93 ± 0.8	3 ± 0.76	26.57	** V1 < V3, ** V1 < V4, ** V2 < V4, ** V3 < V4
	3	1 ± 0	1.07 ± 0.26	1.53 ± 0.99	2.13 ± 0.99	8.11	** V1 < V4, ** V2 < V4
	4	1 ± 0	1 ± 0	1.2 ± 0.56	1.4 ± 0.83	2.2	ns
	5	1.07 ± 0.26	1.4 ± 0.51	1.87 ± 0.99	2.53 ± 1.06	9.98	* V1 < V3, ** V1 < V4, ** V2 < V4
	Total	5.07 ± 0.26	6.27 ± 1.28	7.87 ± 3.16	11.23 ± 3.24	19.58	** V1 < V4, * V1 < V3
+15 cm	1	1 ± 0	1.27 ± 0.46	1.4 ± 0.51	2.33 ± 1.11	11.84	** V1 < V4, ** V2 < V4, ** V3 < V4
	2	1 ± 0	1.6 ± 0.51	1.87 ± 0.74	2.87 ± 0.83	24.16	** V1 < V3, ** V1 < V4, ** V2 < V4, ** V3 < V4
	3	1 ± 0	1.2 ± 0.41	1.67 ± 0.82	2.13 ± 0.92	9.17	* V1 < V3, ** V1 < V4, ** V2 < V4
	4	1 ± 0	1.1 ± 0.26	1.27 ± 0.59	1.27 ± 0.46	1.8	ns
	5	1 ± 0	1.6 ± 0.74	2 ± 0.93	2.53 ± 1.06	9.96	** V1 < V3, ** V1 < V4, * V2 < V4, * V2 < V4
	Total	5 ± 0	6.73 ± 1.58	8.2 ± 2.76	11.13 ± 3.29	19.34	** V1 < V4, * V1 < V3, ** V2 < V4, * V3 < V4

## Data Availability

The data supporting the findings of this study are available from the corresponding author upon reasonable request.
